# Expression Patterns of *Claudin* Family Members During Tooth Development and the Role of Claudin-10 (*Cldn10*) in Cytodifferentiation of Stratum Intermedium

**DOI:** 10.3389/fcell.2020.595593

**Published:** 2020-10-22

**Authors:** Xin Wang, Yuta Chiba, Lingling Jia, Keigo Yoshizaki, Kan Saito, Aya Yamada, Man Qin, Satoshi Fukumoto

**Affiliations:** ^1^Department of Pediatric Dentistry, Peking University School and Hospital of Stomatology, Beijing, China; ^2^Division of Pediatric Dentistry, Department of Oral Health and Development Sciences, Tohoku University Graduate School of Dentistry, Sendai, Japan; ^3^State Key Laboratory of Oral Diseases, National Clinical Research Center for Oral Diseases, Department of Cariology and Endodontics, West China Hospital of Stomatology, Sichuan University, Chengdu, China; ^4^Section of Orthodontics and Dentofacial Orthopedics, Division of Oral Health, Growth and Development, Faculty of Dental Science, Kyushu University, Fukuoka, Japan; ^5^Section of Oral Medicine for Children, Division of Oral Health, Growth and Development, Faculty of Dental Science, Kyushu University, Fukuoka, Japan

**Keywords:** tooth development, enamel, stratum intermedium, tight junction, claudin-10, cell differentiation, alkaline phosphatase

## Abstract

There is growing evidence showing that tight junctions play an important role in developing enamel. Claudins are one of the main components of tight junctions and may have pivotal functions in modulating various cellular events, such as regulating cell differentiation and proliferation. Mutations in *CLDN10* of humans are associated with HELIX syndrome and cause enamel defects. However, current knowledge regarding the expression patterns of claudins and the function of *Cldn10* during tooth development remains fragmented. In this study, we aimed to analyze the expression patterns of claudin family members during tooth development and to investigate the role of *Cldn10* in amelogenesis. Using cap analysis gene expression of developing mouse tooth germs compared with that of the whole body, we found that *Cldn1* and *Cldn10* were highly expressed in the tooth. Furthermore, single-cell RNA-sequence analysis using 7-day postnatal Krt14-RFP mouse incisors revealed *Cldn1* and *Cldn10* exhibited distinct expression patterns. *Cldn10* has two isoforms, *Cldn10a* and *Cldn10b*, but only *Cldn10b* was expressed in the tooth. Immunostaining of developing tooth germs revealed claudin-10 was highly expressed in the inner enamel epithelium and stratum intermedium. We also found that overexpression of *Cldn10* in the dental epithelial cell line, SF2, induced alkaline phosphatase (*Alpl*) expression, a marker of maturated stratum intermedium. Our findings suggest that *Cldn10* may be a novel stratum intermedium marker and might play a role in cytodifferentiation of stratum intermedium.

## Introduction

Ectodermal organs, such as teeth, hair follicles, mammary glands, lung and kidney, share initial pattern of development, despite their ultimate diversity in form and function. All the ectodermal organs require regulation of reciprocal and inductive interactions between epithelial cells derived from the ectoderm and mesenchymal cells derived from the neural crest or mesoderm (Thesleff and Sharpe, 1997). Tooth morphogenesis is a typical process that requires sequential and reciprocal signals transmitted between dental epithelium and mesenchyme (Soukup et al., 2008). In human tooth and mouse molar development, the teeth proceed chronologically through four morphological stages—the initiation stage, bud stage, cap stage, and bell stage (Thesleff and Sharpe, 1997). The initiation stage of tooth morphogenesis arises in the oral ectoderm, causing thickening of the dental lamina, and then grows into the mesenchyme to form a tooth bud. This is followed by the cap stage in which the dental epithelium continues to extend into the enclosed mesenchyme to form a transient primary enamel knot. Subsequent to the cap stage is the bell stage when the dental epithelial stem cells further differentiate into enamel epithelium as four distinct cell layers with different structural and physiological characteristics. These layers are composed of inner enamel epithelium cells (IEE), which later differentiate into ameloblasts, stratum intermedium (SI) cells, stellate reticulum (SR) cells, and outer enamel epithelium (OEE) cells. These differentiation processes of dental epithelium are strictly regulated by spatiotemporal expression of genes. We have previously identified several molecules that are essential for tooth development processes (Nakamura et al., 2008; Liu J. et al., 2016; Miyazaki et al., 2016; Arai et al., 2017; Han et al., 2018; He et al., 2019; Chiba et al., 2020; Saito et al., 2020a).

Enamel formation is a unique biomineralization process. Enamel is distinct from other mineralized tissues as its scaffold enamel matrices are produced by ameloblasts, which have an epithelial origin. Developing ameloblasts progress through four stages—the inductive stage, secretory stage, transition stage, and maturation stage (Thesleff and Sharpe, 1997). The onset of secretion of enamel matrix proteins starts after pre-ameloblasts transform into secretory-stage ameloblasts by elongating into tall columnar cells with Tomes’ processes at their apical ends nearest the forming enamel. The key structural enamel matrices (and their genes) are amelogenin (*Amelx*), ameloblastin (*Ambn*), and enamelin (*Enam*). By the end of the secretory stage, the enamel layer achieves its full thickness, but is only partially mineralized. The ameloblasts mark the transition into the maturation stage when they retract their Tomes’ processes and become shorter and more squat-shaped. At the secretory stage, ameloblasts actively secrete the early protease, matrix metalloproteinase-20 (*Mmp-20*). *Mmp-20* slowly degrades enamel proteins, which allows the crystallites to grow in width and thickness so as to fill up the space yielded by the lost proteins. During the transition and maturation stages, ameloblasts secrete kallikrein-related peptidase-4 (*Klk4*) as the late protease to aggressively cleave the residual enamel proteins and remove organic matrices from enamel (Bartlett and Simmer, 1999; Lu et al., 2008). Moreover, ameloblasts require coordination with SI cells to accomplish the mineralization of enamel (Lacruz et al., 2013; McKee et al., 2013).

The SI cells emerge during the bell stage of tooth development and lie on the proximal side of the IEE or ameloblasts as one- to three-layered rows of cuboidal cells until the end of amelogenesis (Miyazaki et al., 2016). SI cells possess dynamic and interactive properties and help ameloblasts to differentiate via the sonic hedgehog (*Shh*) signaling (Koyama et al., 2001). During the later stages of tooth development, differentiated SI cells produce high amounts of alkaline phosphatase (*Alpl*), which promotes enamel mineralization. These findings suggest that SI cells play an important role in enamel mineralization in concert with ameloblasts (Bonting and Nuki, 1963). Previous studies have shown that *Notch1* is highly expressed in SI cells (Mitsiadis et al., 1998). Cell fate determination of SI cells in the enamel organ might be regulated by Notch signaling (Mitsiadis et al., 2005; Harada et al., 2006; Cai et al., 2011).

Tight junctions (TJs) form barriers at the apices of junctional complexes connecting epithelial or endothelial cells. In addition to their classical roles in generating cell polarity and regulating paracellular ion transport, they also provide signaling input for a wide variety of cellular events (Zihni et al., 2016; Hagen, 2017). Accumulating evidence demonstrates that TJs are essential for enamel formation and are located at both the proximal and distal ends of ameloblasts during the secretory and maturation stages of development (Sasaki et al., 1984; Hata et al., 2010). The SI cells are connected to one another by TJs, as well as to the SR cells and ameloblasts (Sasaki et al., 1984).

Claudins are principal barrier-forming proteins that form the backbones of TJs. They are small proteins with four transmembrane segments and two extracellular domains. The claudin family consists of 26 known members in humans and 27 known members in rodents and are expressed in a tissue-specific manner (Tsukita et al., 2019). In some tissues, a single claudin may be both sufficient and essential to form TJs (Gow et al., 1999). On the contrary, some organs require different claudin family members to co-assemble into one TJ complex (Furuse et al., 1999). It is now widely accepted that each claudin has its own unique molecular properties in terms of charge, selectivity, and their roles in signal transduction (Miwa et al., 2001; Van Itallie and Anderson, 2006; Zhang X. et al., 2019).

*Cldn10* encodes the claudin-10 protein and has two main alternatively spliced variants, *Cldn10a* and *Cldn10b*, which differ in their first exon. *Cldn10a* is exclusively expressed in the kidney, whereas *Cldn10b* is detectable in many tissues, including the kidney, skin, salivary glands, brain, lung, and pancreas. Although both isoforms are highly expressed in the kidney, the expression of claudin-10a is concentrated in the cortex, whereas claudin-10b is enriched in the medulla (Milatz and Breiderhoff, 2017). These two variants differ in their selective regulation of ion permeability. Claudin-10a forms a selective paracellular anion channel, whereas claudin-10b acts as a selective cation channel (Milatz and Breiderhoff, 2017). Humans with a homozygous *CLDN10b* variant, N48K, have pathogenic consequences, such as inability to sweat, abnormal cation reabsorption, hypermagnesemia, and kidney damage as a result of perturbed paracellular Na^+^ selectivity (Klar et al., 2017). Also, in a recent study it was shown that mutations in *CLDN10b* of humans are associated with HELIX syndrome and cause dysfunction in TJs leading to abnormalities in renal ion transport, ectodermal gland homeostasis, and epidermal integrity (Hadj-Rabia et al., 2018). In addition, patients with HELIX syndrome show enamel defects, indicating that *Cldn10* may play a role in enamel formation.

Although the claudin family includes a large number of members and claudins are a major component of TJs, their expression patterns are poorly understood. Furthermore, knowledge regarding the spatiotemporal expression of *Cldn10* during tooth morphogenesis is fragmentary and incomplete. In the present study, we aimed to investigate the expression patterns of the claudin family genes and the role of *Cldn10* during tooth development.

## Materials and Methods

### Animal Experiments

The Tg(KRT14-RFP)#Efu mouse line (Krt14-RFP) was obtained from Dr. Matthew P. Hoffman (National Institutes of Health, National Institute of Dental and Craniofacial Research, Bethesda). The animal experiments and protocols were approved by the Tohoku University Animal Care Committee (Animal Protocol number 2020DnA-016-01) and all the animals were housed in the Institution for Animal Experimentation Tohoku University Graduate School of Medicine.

### Single-Cell RNA Sequencing (scRNA-seq) Analysis

Single-cell library preparation, sequencing, and data processing were performed as previously described (Chiba et al., 2020). A total of 6,260 cells from the incisors of postnatal (P) day P7 Krt14-RFP mice were included for subsequent clustering evaluation. Secondary analysis and filtering were performed using the Seurat v3 R package (Stuart et al., 2019). To assign epithelial and mesenchymal cells to distinct clusters based on differentially expressed gene transcripts, significant dimensions were first defined using the principal component analysis (PCA). The number of significant principal components (PCs) for clustering analysis was determined using the “JackStraw” function of the Seurat package at *p* < 0.05 with 15 PCs. The significant PCs were included for graph-based clustering using the Seurat “findClusters” function. Cluster representations were generated using Uniform Manifold Approximation and Projection (UMAP) at a resolution of 0.2.

### Cap Analysis Gene Expression (CAGE)

For CAGE, total RNA was isolated from mouse tooth germs and whole body using TRIzol Reagent (Life Technologies, Carlsbad, CA, United States) and purified using an RNeasy Mini Kit (Qiagen, Hilden, Germany), according to the manufacturers’ protocols. RNA quality was verified using an Agilent Bioanalyzer System (Agilent, Pal Alto, CA, United States), with the results revealing an RNA integrity number (RIN) >8.5 for all the samples. CAGE was performed by DNAFORM (Yokohama, Japan) as previously described (Funada et al., 2020).

### Cell Culture and Transfection

The rat incisor-derived dental epithelial cell line, SF2, was cultured in Dulbecco’s modified Eagle’s medium (DMEM)/F-12 (Invitrogen, Carlsbad, CA, United States) supplemented with 10% fetal bovine serum (FBS), 100 units/mL penicillin and streptomycin in a humidified atmosphere containing 5% CO_2_ at 37°C (Nakamura et al., 2016). Mouse dental papilla (mDP) cells were cultured in DMEM (Invitrogen) supplemented with 10% FBS as described above. For the transfection experiments, SF2 cells were transfected with the *Cldn10*-transcript variant b expression vector pCMV6-Entry-*Cldn10b* (OriGene Technologies, Rockville, MD, United States). The pCMV6-Entry vector was used as a mock control. For transfection, Lipofectamine LTX with Plus reagent (Invitrogen) and plasmid DNA were diluted in Opti-MEM (Gibco, Gaithersburg, MD, United States), mixed at room temperature for 20 min, and then added to the cultures according to the manufacturer’s instructions. After 24 h of transfection, the medium was replaced. Cells were harvested and analyzed at 24, 48, and 72 h post-transfection.

### Reverse Transcription-Polymerase Chain Reaction (RT-PCR) and Quantitative RT-PCR (RT-qPCR)

P7 rat tissue specimens and developing mouse tooth germs were dissected and their total RNA was extracted using RNeasy Kit (Qiagen). SF2 cells were plated into 35-mm dishes at 2 × 10^5^ cells/dish. At 24 h post-transfection, total RNA was isolated from the cells or tissues using an RNeasy Kit (Qiagen), according to the manufacturer’s instructions. The synthesis of complementary DNA (cDNA) was performed in a 20 μL reaction mixture containing 1 μg total RNA at 45°C for 50 min using SuperScript^TM^ VILO^TM^ Master Mix (Invitrogen). RT-qPCR was performed using SYBR Select Master Mix (Applied Biosystems, CA, United States) and the ABI Step-One Real-Time PCR System (Applied Biosystems). The annealing temperature was 61°C. The mRNA expression levels were normalized to that of *Gapdh*. Sequences of the primers used in the study are shown in Table 1.

**TABLE 1:** RT-PCR and RT-qPCR primers used in the current study.

Genes	Product size (bp)	Primers	Sequences (5^*mrGapdh*^
*mrGapdh*	179	Forward	GGTGAAGGTCGGTGTGAACG
		Reverse	CTCGCTCCTGGAAGATGGTG
*mrCldn10*	145	Forward	ATCCAGGCGTGTAGAGGACT
		Reverse	ATACAACCCCAGCCAAGCAA
*rCldn10–a*	127	Forward	TAACCGCCACCTGGGTTTAC
		Reverse	AGTCCTCTACACGCCTGGAT
*rCldn10–b*	131	Forward	GTCATCACCACCGCCACTTA
		Reverse	AGTCCTCTACACGCCTGGAT
*mrCldn1*	459	Forward	GCCATCTACGAGGGACTGTG
		Reverse	TACCATCAAGGCTCKGGTTG
*rAlpl*	101	Forward	GGGTGGGTTTCTCTCTTGGG
		Reverse	ATGATGGTTGCAGGGTCTGG
*mrAmbn*	137	Forward	TGTAGGTCCCTTCTTGCTTCC
		Reverse	TGCCTAAGACAGCTACATGCT
*mrAmeloD*	151	Forward	TTACGACTACCCGTTCGAGC
		Reverse	CAGTGTCTCCACCTTGCTGA
*mrNotch1*	105	Forward	TCCTGAAGAACGGAGCCAAC
		Reverse	CCAGCAACACTTTGGCAGTC

**RT–PCR, reverse transcription polymerase chain reaction; RT–qPCR, quantitation RT–PCR; bp, base pair; m, mouse; r, rat.**

### Immunofluorescence Staining

For cell immunofluorescence staining, transiently transfected SF2 cells were grown on glass coverslips and fixed in 4% paraformaldehyde at 48 h after transfection. Cells were washed three times with PBS. After blocking with 1% bovine serum albumin, the cells were incubated with the claudin–10 (1:100; Invitrogen) and ZO–1 (1:500; Cell Signaling, Beverly, MA, United States) primary antibodies, followed by incubation with the Alexa–488–labeled secondary antibody (1:400; Invitrogen) and Hoechst 33342 nuclear stain (1:1,500).

For immunofluorescence staining of the tooth germ, the heads of mice were collected on embryonic (E) day E13, E14, and E16, and P1. The heads were fixed in 4% paraformaldehyde for 24 h, dehydrated in a gradient concentration of ethanol, and then embedded in paraffin. P3 mouse heads were also collected and decalcified in 15% EDTA for 2 week prior to dehydration and paraffin embedding. The embedded specimens were sectioned at 6 μm thickness, deparaffinized, rehydrated, and microwaved in citrate buffer (Agilent) for antigen retrieval. The sections were subsequently incubated in Power Block^TM^ Universal Blocking Reagent (BioGenex, Fremont, CA, United States) for 10 min, followed by overnight incubation at 4°C with the claudin-10 primary antibody. After incubation with the primary antibody, the slides were treated with the Alexa-488-labeled secondary antibody (1:400; Invitrogen) and Hoechst 33342 nuclear stain (1:1,500) for 40 min. Images were captured using a Fluoview FV10i confocal laser-scanning microscope system (Olympus Life Science, Tokyo, Japan).

### Western Blot Analysis

SF2 cells were plated in 60 mm dishes at 4 × 10^5^ cells/dish. At 48 h post-transfection, the cells were washed twice with ice-cold phosphate-buffered saline containing 1 mM sodium vanadate (Na_3_VO_4_) and then lysed at 4°C for 15 min in 50 μL EBC lysis buffer [50 mM Tris (pH 8.0), 120 mM NaCl, 0.5% NP-40] supplemented with complete protease inhibitor cocktail (Roche Molecular Biochemicals, Mannheim, Germany). Protein concentrations were determined using the Micro-BCA Protein Assay Reagent (Thermo Fisher Scientific, Waltham, MA, United States). Aliquots of protein extracts (25 μg per sample) were subjected to 4–12% sodium dodecyl sulfate (SDS)-polyacrylamide gel electrophoresis (PAGE) and transferred to polyvinylidene fluoride (PVDF) membranes. The membranes were blocked with 3% bovine serum albumin (Sigma-Aldrich, St Louis, MO, United States) for 1 h and then incubated overnight with the claudin-10 primary antibody (1:1,000; Invitrogen) at 4°C. Antibody binding signals were detected using a horseradish peroxidase-conjugated secondary antibody (Cell Signaling) and the SuperSignal West Dura Substrate Kit (Thermo Fisher Scientific), as previously described (Saito et al., 2020b).

### Cell Proliferation Assay

SF2 cells were seeded in 96-well plates at 6 × 10^3^ cells/well. At day 0, 1, and 2 post-transfection, cell proliferation activity was measured using the Cell Counting Kit-8 (CCK-8; Dojindo, Japan). After incubation at 37°C for 1 h in complete medium with the CCK-8 reagent, absorbance at 450 nm was measured using a TriStar^2^ LB 942 microplate reader (Berthold Technologies, Bad Wildbad, Germany).

### Statistical Analysis

The data are presented as means ± standard deviation (SD). Comparisons were performed using the GraphPrism6 statistical software (GraphPad Software, United States). A two-tailed Student’s *t*-test was applied for comparison of two independent variables. Multiple analysis of the CCK-8 assay results was conducted using two-way analysis of variance (ANOVA). Differences were considered statistically significant for *p* < 0.05.

## Results

### Transcriptome Analysis Revealed Gene Expression Profiles of Claudin Family Members

**FIGURE 1:**
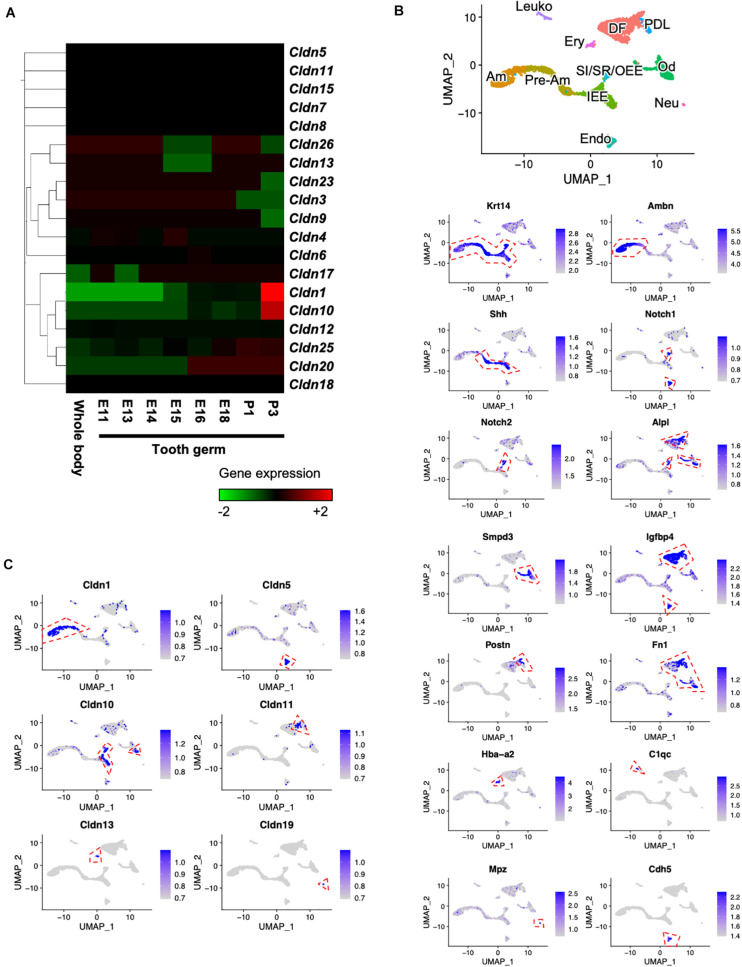
Expression patterns of claudin family members during tooth development. **(A)** Heatmap of Cap Analysis Gene Expression (CAGE)-based claudin genes expression in embryonic (E) day E11 mouse whole body and tooth germs at various developmental stages. **(B)** Differential expression analysis of cell-type marker genes using Uniform Manifold Approximation and Projection (UMAP) visualization of single-cell datasets from postnatal (P) day P7 Krt14-RFP mouse incisors. *Krt14*, epithelium marker; *Ambn*, ameloblast marker; *Shh*, IEE and pre-ameloblast marker; *Notch1* and *Alpl*, SI markers; *Notch2*, SR and OEE marker; *Smpd3*, odontoblast marker; *Igfbp4*, dental follicle and *Postn*, periodontal ligament markers; *Fn1*, mesenchyme marker; *Hba-a2*, erythrocyte marker; *C1qc*, leukocyte marker; *Mpz*, neural cell marker; *Cdh5*, endothelial cell marker. *IEE*, inner enamel epithelium; *Pre-Am*, pre-ameloblast; *Am*, ameloblast; *SI/SR/OEE*, stratum intermedium, stellate reticulum, and outer enamel epithelium; *DF*, dental follicle; *Od*, odontoblast; *PDL*, periodontal ligament; *Ery*, erythrocyte; *Leuko*, leukocyte; *Endo*, endothelium; *Neu*, neural cell. Red dashed lines indicate the cell cluster that highly expressed marker genes. **(C)** Differential gene expression analysis of claudin family members detected in single-cell datasets from P7 Krt14-RFP incisors. Red dashed lines indicate the cell cluster that highly expressed marker genes.

To clarify the gene expression patterns of claudin genes during tooth development, we performed CAGE using total RNA extracted from E11 whole body and E11, E13, E14, E15, E16, E18, P1, and P3 molars. Heatmap analysis of claudin family member genes revealed 19 claudin genes that were expressed (Figure 1A). *Cldn1* and *Cldn10* were highly expressed in P3 tooth germs. We then performed scRNA-seq to determine the expression patterns of the claudin genes at a single-cell level. The scRNA-seq was performed using incisors of P7 Krt14-RFP mice, as previously described (Chiba et al., 2020). Unbiased clustering based on the Seurat v3 algorithm revealed 11 clusters using UMAP (Figure 1B). To characterize the clusters, they were classified by cell type based on known markers for dental epithelium and mesenchyme, which included IEE and pre-ameloblast marker, sonic hedgehog (*Shh*) (Dassule et al., 2000); ameloblast marker, ameloblastin (*Ambn*) (Fukumoto et al., 2004); SI cell markers Notch1 (*Notch1*) and alkaline phosphatase (*Alpl*) (Harada et al., 1999; Lacruz et al., 2013); SR and OEE cell marker, Notch2 (*Notch2*) (Harada et al., 1999; Lacruz et al., 2013); dental follicle marker, insulin-like growth factor binding protein 4 (*Igfbp4*) (Seidel et al., 2017); odontoblast marker, sphingomyelin phosphodiesterase 3 (*Smpd3*) (Khavandgar et al., 2013); periodontal ligament marker, periostin (*Postn*) (Afanador et al., 2005); erythrocyte marker, hemoglobin A2 (*Hba-a2*); leukocyte marker, complement C1q chain (*C1qc*); endothelial cell marker, cadherin 5 (*Cdh5*); and neural cell marker, myelin protein zero (*Mpz*). We then investigated gene expression patterns of claudin family members in these distinct cell clusters. In this scRNA-seq dataset, transcripts of only six claudin family members were detected—*Cldn1* in ameloblasts, *Cldn5* in endothelium, *Cldn10* in IEE and SI/SR/OEE, *Cldn11* in dental follicles, *Cldn13* in erythrocytes, and *Cldn19* in neural cells. *Cldn1* was highly expressed in ameloblasts (Figure 1C), consistent with a previous report (Zhang Y. et al., 2019). Unlike *Cldn1*, *Cldn10* was abundantly expressed in IEE and SI/SR/OEE epithelial cells and expressed in cell clusters representing odontoblasts of mouse incisors (Figure 1C).

### *Cldn10b* Is Highly Expressed in Developing Teeth

**FIGURE 2:**
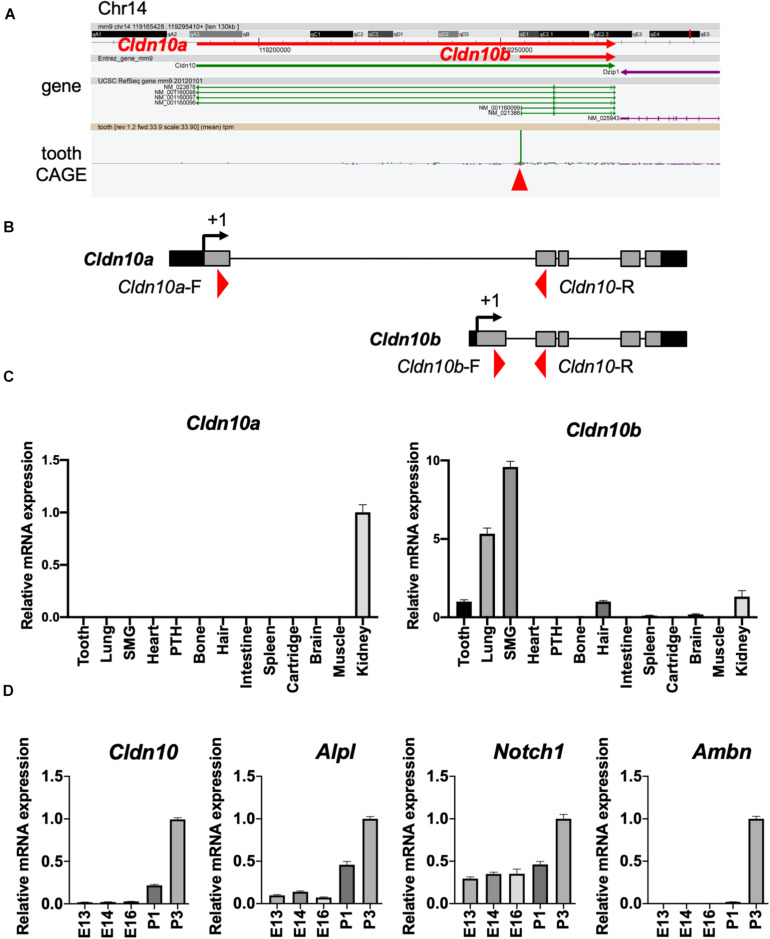
Expression patterns of *Cldn10* during tooth development. **(A)** Genomic locus of mouse *Cldn10* in Cap Analysis Gene Expression (CAGE). The CAGE dataset from tooth germs showed a high peak at the *Cldn10b* transcription start site. The red arrowhead indicates the CAGE peak. **(B)** Schematic summary of *Cldn10* isoforms in the rat genomic locus. Red arrowheads indicate the locations of intron-spanning primers used in quantitative reverse transcription polymerase chain reaction (RT-qPCR). **(C)** Expression levels of *Cldn10* mRNA isoforms a and b in postnatal (P) day P7 rat tissues (*n* = 3). The relative mRNA expression was standardized to *Gapdh* expression. Error bars represent S.D. *SMG*, submandibular gland; *PTH*, parathyroid. **(D)** mRNA expression levels of *Cldn10*, SI cell markers *Alpl* and *Notch1*, and ameloblast marker *Ambn* at different developmental stages of mouse tooth germ (*n* = 3). The relative mRNA expression was standardized to *Gapdh* expression. Error bars represent S.D.

Based on the transcriptome data shown in Figure 1, we focused on *Cldn10* as a key molecule for enamel formation. *Cldn10* may be expressed as two splice variants, *Cldn10a* and *Cldn10b*, which exhibit different expression patterns in organs. Utilizing the CAGE data, tooth-specific transcriptional start sites were explored, as previously described (Funada et al., 2020). The CAGE dataset obtained from mouse tooth germs revealed that only *Cldn10b* showed a high peak in tooth samples (Figure 2A). We also analyzed the expression of *Cldn10a* and *Cldn10b* in P7 rat tissue specimens using RT-qPCR and primers specific for each splice variant (Figure 2B). RT-qPCR results indicated that *Cldn10a* expression was restricted to the kidney, whereas *Cldn10b* expression was detected at relatively high levels in the ectodermal organs evaluated, including tooth, lung, submandibular gland, hair, and kidney (Figure 2C). Therefore, *Cldn10b* apparently may play a role in tooth development. Next, we investigated the expression patterns of *Cldn10* in the tooth germs at various stages using dental epithelial marker genes. *Alpl* and *Notch1* are known to be the markers of SI cells in the dental epithelium and *Ambn* is an enamel matrix marker for ameloblasts. RT-qPCR analysis showed that *Alpl* shared a similar expression pattern with *Cldn10b* and both were strongly expressed during later stages of tooth development (Figure 2D). In contrast, *Notch1* was detected at relatively constant levels throughout dental development with an elevation at P3 (Figure 2D) whereas *Ambn* was highly expressed at P3 when secretory ameloblasts were actively functioning (Figure 2D).

### Claudin-10 Localizes to IEE and SI Cells in Developing Teeth

**FIGURE 3:**
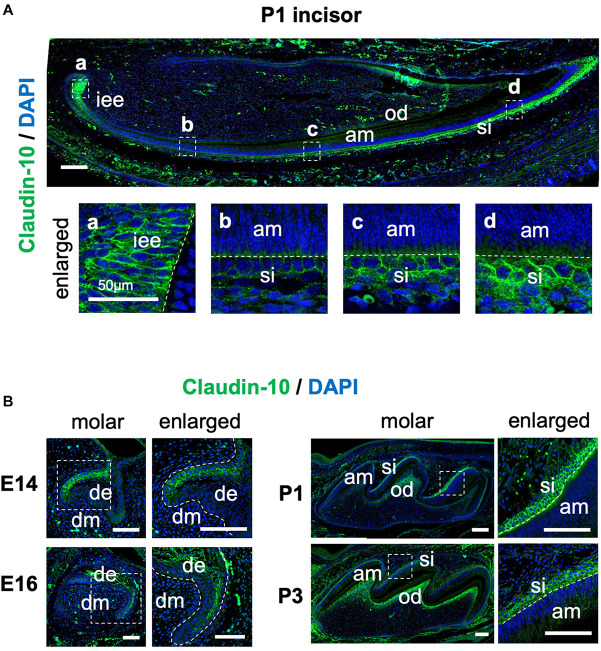
Spatiotemporal expression pattern of claudin-10 during tooth development. **(A)** Immunofluorescence staining of claudin-10 in postnatal (P) day P1 mouse lower incisors. Dashed white boxes indicate the area enlarged in the lower panel. Scale bar, 100 μm. *Lower panel:* enlarged image shown in the upper panel. Scale bars, 50 μm. **(B)** Immunofluorescence staining of claudin-10 in embryonic (E) day E14, E16, P1, and P3 mouse molar tooth germs. *Green*, claudin-10; *blue*, DAPI (4’,6-diamidino-2-phenylindol); *iee*, inner enamel epithelium; *si*, stratum intermedium; *am*, ameloblast; *od*, odontoblast; *de*, dental epithelium; *dm*, dental mesenchyme. Dashed white boxes indicate the area enlarged in the right panel. Scale bars, 100 μm.

To examine the spatiotemporal protein expression of claudin-10 in developing teeth, we performed immunofluorescence staining using sections of mouse P1 lower incisor and E14, E16, P1, and P3 molars. In the incisors, claudin-10 expression was high in IEE and SI cells during the secretory stage and in differentiated odontoblasts (Figure 3A). From the proximal to the distal parts of the P1 incisor, claudin-10 expression was restricted to SI cells in a gradually increasing manner and had the highest expression level at the incisor tip. For molars, claudin-10 expression was initially localized in the lingual dental epithelium of E14 tooth germs, which is consistent with a previous report (Kim et al., 2020). In E16 molars, claudin-10 was found to be broadly expressed in both SR and labial IEE cells (Figure 3B). For P1 and P3 tooth germs, claudin-10 was mainly and stably expressed in SI cells and in the odontoblastic layer (Figure 3B).

### Overexpression of *Cldn10b* Promotes *Alpl* Expression in the Dental Epithelium

**FIGURE 4:**
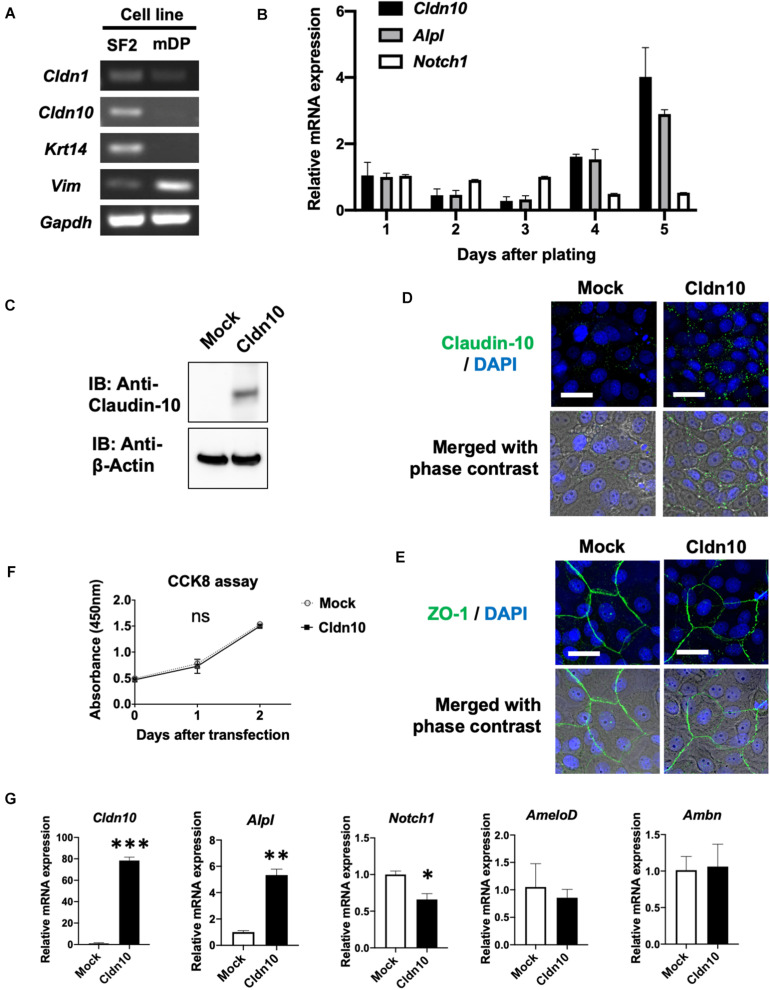
Overexpression of claudin-10b enhances differentiation of rat incisor-derived dental epithelial SF2 cells into the stratum intermedium (SI) cell lineage. **(A)** Reverse transcription polymerase chain reaction (RT-PCR) analysis of *Cldn1*, *Cldn10*, *Krt14*, and *Vim* expression in SF2 cells and a mouse dental papilla (mDP) mesenchymal cell line. *Krt14* and *Vim* were used as representative epithelial and mesenchymal marker genes, respectively. **(B)** Time-course analysis of *Cldn10*, *Alpl*, and *Notch1* expression in SF2 cells using quantitative RT-qPCR (*n* = 3). Relative mRNA expression was standardized to *Gapdh* expression. Error bars represent S.D. **(C)** SF2 cells were transfected with a mock control vector or a *Cldn10b* expression vector. Western blot analysis showed high expression levels of claudin-10 protein in *Cldn10b-*overexpressing SF2 cells. **(D)** Immunofluorescence of Claudin-10 in mock-transfected and *Cldn10b-*overexpressing SF2 cells. Upper panel: *Green*, claudin-10; *blue*, DAPI. Lower panel: merged image with phase contrast from upper panel. Scale bars, 10 μm. **(E)** Immunofluorescence of ZO-1 in mock-transfected and *Cldn10b-*overexpressing SF2 cells. Upper panel: *Green*, ZO-1; *blue*, DAPI. Lower panel: merged image with phase contrast from upper panel. Scale bars, 10 μm. **(F)** Cell Counting Kit-8 (CCK-8) assay of mock-transfected and *Cldn10b*-transfected cells revealed similar proliferation rates (*n* = 3). Error bars represent S.D. ns (not significant), *p* > 0.05 with two-way ANOVA. **(G)** Expression of *Alpl*, *Notch1*, *AmeloD*, and *Ambn* in *Cldn10b*-overexpressing SF2 cells (*n* = 3). Error bars represent S.D. ^∗^*p* < 0.05; ^∗∗^*p* < 0.01; ^∗∗∗^*p* < 0.001 with two-tailed *t*-test.

To further analyze *Cldn10*, we used an *in vitro* cell culture system. Expression of *Cldn1* and *Cldn10* was examined in the rat dental epithelial cell line, SF2, and mouse dental mesenchymal cell line, mDP (Figure 4A). As expected, *Cldn1* and *Cldn10* were primarily expressed in dental epithelial cells with *Cldn10* expression being absent in dental mesenchymal cells (Figure 4A). As SF2 cells have characteristics of IEE cells and are able to differentiate into the SI cell lineage (Nakamura et al., 2008; Arakaki et al., 2012), we used SF2 cells to further analyze the role of *Cldn10* in the dental epithelium.

Changes in *Cldn10*, *Alpl*, and *Notch1* expression were examined using time-course culturing of SF2 cells (Figure 4B). RT-qPCR results demonstrated that the expression of *Cldn10* and *Alpl* was upregulated after 4 days of culturing, whereas *Notch1* expression was downregulated. We then analyzed the effects of *Cldn10* overexpression on cell proliferation and differentiation. As the specific expression of *Cldn10b*, and not of *Cldn10a*, was observed in the developing teeth, we used a claudin-10b expression vector for analysis. We first examined protein levels of claudin-10b in mock-control and *Cldn10b*-overexpressing SF2 cells (Figure 4C). In *Cldn10b*-overexpressing SF2 cells, claudin-10 protein was highly expressed, although the expression of endogenous claudin-10 was not clearly detected in mock-control SF2 cells (Figure 4C). We performed immunofluorescence analysis of claudin-10 and the tight junction molecule, ZO-1, in SF2 cells to examine the TJ formation in this cell line (Figures 4D,E). Claudin-10 was mainly localized in the cytoplasm and in cell–cell junctions in both of mock-control and *Cldn10b*-overexpressing SF2 cells, with the higher level of expression in the latter. ZO-1 was expressed in cell–cell junctions of SF2 cells, suggesting that SF2 form TJ and claudin-10 may contribute in TJ formation. We then analyzed the effect of *Cldn10b* on cell proliferation and found that overexpression of *Cldn10b* in SF2 cells had no effect on cell proliferation compared with that of transfected mock control SF2 cells (Figure 4F). We further examined the effect of *Cldn10b* on cytodifferentiation using RT-qPCR. *Cldn10b*-overexpressing SF2 cells showed a significant upregulation of *Alpl* expression, whereas *Notch1* expression was downregulated (Figure 4G). Expression of the IEE marker gene, *AmeloD* (Chiba et al., 2019; He et al., 2019), and ameloblast marker gene, *Ambn*, did not exhibit any notable changes (Figure 4G). These results suggest that *Cldn10b* promotes SI cell differentiation in dental epithelium.

## Discussion

In this study, we investigated the gene expression patterns of claudin family members during tooth development and examined the role of *Cldn10* in SI cells. Previous studies have shown that *Cldn10* is expressed in mouse dental epithelium and SI cells (Ohazama and Sharpe, 2007; Kim et al., 2020); however, a comprehensive evaluation of spatiotemporal expression pattern of *Cldn10* during amelogenesis has not been reported. Our immunostaining results revealed that claudin-10 was expressed in IEE and SI cells and expression levels gradually increased in the later stages of tooth development (Figures 3A,B). Furthermore, *Cldn10* and *Alpl* exhibited similar expression patterns, including high expressing in SI cells during later stages of tooth development, suggesting an association between the expression of *Cldn10* and *Alpl* (Figures 2D, 4B). Subsequent functional analysis revealed *Cldn10*-induced *Alpl* expression (Figure 4G). These findings suggest that *Cldn10* promotes cytodifferentiation of SI cells.

Claudins, as key components of TJs, are essential for cellular events, such as cell signaling, formation of the paracellular barrier, and regulation of the epithelial permeability (Barrios-Rodiles et al., 2005). Variable expression of claudins in different cells and tissues of the body suggests that each claudin member has a distinct function. Although TJs are broadly distributed in the dental epithelium, only a few claudins have been investigated and proven to play the role in tooth development (Ohazama and Sharpe, 2007; Hagen, 2017; Kim et al., 2020). Mutations in *CLDN10*, *CLDN16*, and *CLDN19* are associated with amelogenesis imperfecta in humans (Bardet et al., 2016; Yamaguti et al., 2017; Kim et al., 2020) and in mice, deletion of *Cldn3* significantly reduces the enamel volume compared to that of wild-type mice (Bardet et al., 2017). To date, 10 members of the claudin family have been reported to be expressed in the developing teeth. Claudin-1 and claudin-7 are found to have strong immunoreactivity in IEE, OEE, SI, SR, and ameloblasts, whereas claudin-5 is preferentially expressed in only vascular structures (Soukup et al., 2008). Claudin-3, claudin-4, claudin-6, and claudin-7 are expressed in the dental epithelium during early developmental stages, whereas claudin-11 is mainly expressed in the dental follicle (Ohazama and Sharpe, 2007). Claudin-16 and claudin-19 can also be detected in differentiating mouse ameloblasts, corresponding to the phenotypes of patients with *CLDN16* and *CLDN19* mutations (Bardet et al., 2016; Yamaguti et al., 2017; Kim et al., 2020). Our current scRNA-seq analysis demonstrated expression profiles of *Cldn1*, *Cldn5*, *Cldn10*, *Cldn11*, and *Cldn19*, consistent with these previous findings (Figure 1C); however, we did not detect gene transcripts of other claudin family members in our scRNA-seq dataset. Although we obtained 2,386 mean genes per cell in our scRNA-seq dataset, the sequencing saturation was only 50% (Chiba et al., 2020). Therefore, the scRNA-seq read depth may have affected our analysis and resulted in this limited detection of genes.

Transcriptome analysis showed that among the claudin family members, *Cldn1* and *Cldn10* were highly expressed in the tooth germs (Figures 1A,C). Furthermore, immunostaining of claudin-10 demonstrated specific expression in IEE and SI cells. In P1 incisors, claudin-10 was expressed in IEE and SI throughout the proliferation and secretory stages of tooth development. Claudin-10 was also mainly expressed in the SI layer of P1 and P3 molars. Based on these findings, we suggest that *Cldn10* may be a marker gene for SI cells. The origin of the SI cells could be IEE cells, same as the origin of ameloblasts (Harada et al., 2006). In a previous study using rat-incisor organ cultures, it was determined that SI cells are derived from the IEE during the transit-amplifying process (Harada et al., 2006). In the present study, the expression of claudin-10 was continuously observed during the transition of IEE to SI cells (Figure 3A), supporting the hypothesis that IEE cells are able to differentiate into SI cells.

The enamel organ is composed of a highly multifaceted epithelial cell population, all of which is uniquely differentiated toward a specialized function. Among these cell types, SI cells contribute to ameloblast differentiation and promote enamel mineralization through their high alkaline phosphatase activity (Larmas and Thesleff, 1980). In a previous study, high levels of *Alpl* expression were observed in the SI layer, whereas the expression of *Alpl* was completely absent in neighboring SR cells and ameloblasts (Liu H. et al., 2016). The immortalized dental epithelial cell line, SF2, is able to differentiate into both ameloblasts and the SI lineage (Nakamura et al., 2008; Arakaki et al., 2012). Differentiation of SF2 cells into the SI lineage can be indicated by increased *Alpl* expression over time under no intervention (Yoshizaki et al., 2017), which is consistent with our current results (Figure 4B). Considering the distinct expression of claudin-10 in IEE cells prior to SI cell differentiation, we hypothesized a potential involvement of claudin-10 in the differentiation of SI cells. *In vitro* experiments revealed that the overexpression of *Cldn10* in SF2 cells significantly induced *Alpl* expression, although the expression of ameloblast marker genes was not affected (Figure 4G). These results suggest that *Cldn10* might promote the differentiation of SI cells and enamel mineralization through the regulation of *Alpl* expression. Interestingly, *Cldn10* and *Alpl* were colocalized in the odontoblast cell cluster in the scRNA-seq dataset (Figures 1B,C) and immunofluorescence showed that *Cldn10* was localized in odontoblasts (Figure 3). Several reports suggest that odontoblasts form tight junction between their cell-cell junction (Tjäderhane et al., 2013), therefore *Cldn10* might play important roles in odontoblast development processes.

As previously noted, *Cldn10* has two isoforms that differ not only in their patterns of expression, but also in their functions as paracellular channels for anions or cations. *Cldn10a* is preferentially expressed in the kidney, whereas *Cldn10b* is more ubiquitously detected in ectodermal organs, including the tooth germs (Figures 2A,C). Compared to claudin-10a, which forms a selective paracellular anion channel, claudin-10b serves in a paracellular pathway for small cations (Milatz and Breiderhoff, 2017). Although the physiological role of claudin-10a in the proximal tubule is still poorly understood, claudin-10b has been proved to be an important component in renal ion transport, and participates in the regulation of homeostasis of the ectodermal gland (Klar et al., 2017; Hadj-Rabia et al., 2018). Our study shows, for the first time, that *Cldn10b*, and not *Cldn10a*, is expressed in mouse teeth at levels comparable to its expression in the kidney and hair. Mice lacking claudin-10b exhibit disturbances in the homeostasis of Ca^2+^ and Mg^2+^ in the kidney, giving rise to symptoms, such as hypocalcemia, hypermagnesemia, hyperphosphatemia, and nephrocalcinosis (Breiderhoff et al., 2012). In a previous study, it was reported that the HELIX syndrome in humans is caused by bi-allelic mutations in *CLDN10b* (Hadj-Rabia et al., 2018). Patients with the HELIX syndrome present abnormalities in the kidney, skin, salivary glands, and tooth enamel. Tooth abnormality in the HELIX syndrome is characterized as severe enamel wear after tooth eruption, which is an indication of enamel hypomineralization. However, there is still a lack of sufficient evidence to support the notion that the permeability properties of claudin-10b are critical for its role during amelogenesis. Further analysis is required to determine the molecular mechanisms involved in *Cldn10b* mutations causing enamel hypoplasia in humans.

Overall, our study reveals the spatiotemporal gene expression patterns of claudin family members during murine tooth development. Functional analysis suggests a role for *Cldn10* in regulating the differentiation of dental epithelial cells. These findings contribute toward clarifying the molecular mechanism involved in enamel formation and the pathological mechanism of enamel hypoplasia.

## Data Availability Statement

Publicly available datasets were analyzed in this study. The datasets generated for this study can be found in the NCBI GEO: GSE146855.

## Ethics Statement

The animal study was reviewed and approved by the Tohoku University Animal Care Committee (Animal Protocol number 2020DnA-016-01).

## Author Contributions

XW and YC performed the experiments and wrote the manuscript. LJ and KY participated in the experiments. KS and AY contributed to the data interpretation and revised the manuscript. YC and SF designed the study. MQ and SF revised the manuscript and supervised the study. All authors contributed to the article and approved the submitted version.

## Conflict of Interest

The authors declare that the research was conducted in the absence of any commercial or financial relationships that could be construed as a potential conflict of interest.
